# 

**Published:** 1881-02

**Authors:** 


### CONTENTS:

ART. I.—Proceedings of Maryland and District of Columbia Dental Association. Annual Meeting.—(Continued.)......433
ART. II.—The Literature of Dental Mechanism. By Prof. Hodgkin..................442
ART. III.—A Dentalation. By Dr. J. Washington Clowes........ ...............452
ART. IV.—Pathology and Therapeutics—from a Conservative "New Departure" Standpoint. By F. E. Howard, D. D. S.......................460
ART. V.—Caries of the Superior Maxilla. By Truman W. Brophy, M. D , D. D S.........................470

### EDITORIAL, ETC.

The Proposed Extension of the Cummings' Patent.................................473
Protective Legislation for Dentistry.............................................475
The Board of Visitors of the Balto. College of Dental Surgery..........................476

### BIBLIOGRAPHICAL.

Webster's New Unabridged Dictionary.................................. ........477
Transactions of American Medical Association, Vol. XXXI. 1880..... ...........477
Vick's Illustrated Guide for 1881.......... ...............................478
Anatomical Atlas. (Wilde's.)...............................................478
Horse Teeth............... ..................................................478
Transactions of the American Dental Association, 1880.... . ......................478

### MONTHLY SUMMARY.

Tooth Structure Illustrated..................................................479
Wax Tree.....................................................................479
India Rubber Deterioration...................................................480
Poisoning by Tincture of Arnica .... ...........................................480

